# Hepatitis C infection epidemiology in Mongolia: protocol of a systematic review and meta-analysis

**DOI:** 10.1186/s13643-017-0558-8

**Published:** 2017-08-08

**Authors:** Karima Chaabna, Laith J. Abu-Raddad

**Affiliations:** 1 Infectious Disease Epidemiology Group, Weill Cornell Medicine-Qatar, Cornell University, Qatar Foundation-Education City, P.O. Box 24144, Doha, Qatar; 2 Department of Healthcare Policy and Research, Weill Cornell Medicine, Cornell University, New York, USA; 3 College of Health and Life Sciences, Hamad Bin Khalifa University, Doha, Qatar

**Keywords:** Mongolia, Hepatitis C, HCV, Chronic infection, At-risk populations, Genotype, Epidemiology, Prevalence, Incidence, RNA

## Abstract

**Background:**

Hepatitis C virus (HCV) morbidity appears to be high in Mongolia. Yet, the scale and nature of the infection burden is not well-understood. Our study’s objective is to systematically review and synthetize all available epidemiological data on HCV antibody (Ab) prevalence, ribonucleic acid (RNA) prevalence, incidence, risk factors to HCV exposure, and circulating HCV genotypes/subtypes among different at-risk populations. Additionally, we aim to estimate national population-level HCV-Ab prevalence and the number of HCV chronically infected individuals in the population of Mongolia.

**Methods:**

Our systematic review will be reported based on the items outlined in the Preferred Reporting Items for Systematic Reviews and Meta-Analyses (PRISMA 2009) statement. All reports with primary data collected from surveillance or observational studies on Mongolian populations will be eligible for inclusion if the study sample size is greater than 25. Included reports need to present studies that use biological assay for HCV-Ab ascertainment. We will consider three primary outcomes of interest: HCV-Ab incidence, HCV-Ab prevalence, and HCV genotypes/subtypes among different at-risk populations. In addition, two secondary outcomes of interests will be also collected: HCV RNA prevalence, and unadjusted and/or adjusted statistically significant risk factors for HCV exposure (*p* value ≤0.05). In order to identify relevant reports, we will search PubMed, Embase, and Index Medicus for the Southeast Asian region. Additionally, we will search Mongolian scientific and medical journals not indexed in PubMed or Embase and the archives of Mongolian local conferences. Lastly, the literature search will be supplemented by checking references of the included reports and identified reviews. We will use broad search criteria with no language or time restrictions. Meta-analyses will estimate pooled HCV-Ab prevalence (by at-risk population, sex, age group, and period), and pooled RNA prevalence among HCV-Ab positive individuals in the general population. Age-adjustment of estimates will be conducted.

**Discussion:**

The proposed systematic review and meta-analysis will produce a comprehensive synthesis of HCV epidemiology in Mongolia. The study will provide empirical evidence to inform health policy decision-making, resource allocation, and planning and implementation of relevant public health interventions.

**Systematic review registration:**

We have not registered with PROSPERO

**Electronic supplementary material:**

The online version of this article (doi:10.1186/s13643-017-0558-8) contains supplementary material, which is available to authorized users.

## Background

Mongolia is a lower-middle-income country located in Central Asia and bordered by Russia and China [1]. Thirty-two percent of the total population lives in its capital Ulaanbaatar [2]. Mongolia’s population (3.03 million in 2015 [3]) is relatively young with 33% being under 15 years of age and 3.5% being over 65 years of age [3]. Mongolia is unique as it is one of the most sparsely populated countries in the world with 1.9 people per square kilometer [4]. The very low population density and harsh climate make communication, transport, and economic development challenging thereby complicating health service provision in this country [5]. Furthermore, changes in the health system model from a centralized to a decentralized one affected directly or indirectly population health and the capacity of the health sector [6, 7].

Hepatitis C virus (HCV) morbidity appears to be high in Mongolia. Yet, the scale and nature of the infection burden is not well-understood. The objective of this protocol is to describe the methodology developed for a systematic review and synthesis of all available epidemiological data on HCV antibody (Ab) incidence, HCV-Ab prevalence, ribonucleic acid (RNA) prevalence, risk factors to HCV exposure, and circulating HCV genotypes/subtypes among different at-risk populations. Additionally, we aim to estimate national population-level HCV-Ab prevalence and the number of HCV chronically infected individuals among the general population of Mongolia.

## Methods

Our systematic review will be reported based on the items outlined in the Preferred Reporting Items for Systematic Reviews and Meta-Analyses (PRISMA 2009) statement [8]. The current protocol describes our methodology developed and adapted from a published protocol [9, 10] and based on a series of systematic reviews and meta-analyses on HCV in the countries of the Middle East and North Africa [11–16]. Our protocol is reported following PRISMA for Systematic Review Protocols (PRISMA-P 2015) guidelines [17]. Completed PRISMA-P 2015 checklist can be found in Additional file 1: Table S1.

### Inclusion and exclusion criteria

#### Type of studies

All reports with primary data collected from surveillance, cross-sectional, cohort, or case-control studies will be eligible for inclusion if the study sample size is greater than 25. Case reports, case series, reviews, qualitative studies, editorials, commentaries, letters to editors, author replies, and animal studies will be excluded.

As in earlier reviews [11–16], the word ‘report’ refers to a publication of a surveillance or an observational study such as an article, a country-level report, or a conference abstract that present one or more outcome measures.

#### Study population and setting

Included reports will be those including data on Mongolian populations in Mongolia. Therefore, studies conducted on Mongolian nationals outside of Mongolia will be excluded.

#### Type of exposures

Included reports need to present studies that used biological assays for HCV-Ab ascertainment. Reports will be considered ineligible if the studies are based on self-report, or classification of HCV as non-A non-B hepatitis.

### Primary and secondary outcomes of interest

We consider three primary outcomes of interest: HCV Ab-incidence, HCV-Ab prevalence, and HCV genotypes/subtypes among different at-risk populations. In addition, two secondary outcomes of interest will be also collected: HCV RNA prevalence and unadjusted and/or adjusted statistically significant risk factors for HCV exposure (probability (*p*) value ≤0.05).

### Data sources and search strategy

In order to identify relevant reports, we will search PubMed [18], Embase [19], and the Global Health Library (Index Medicus for the Southeast Asian region) [20]. Additionally, we will search 14 Mongolian scientific and medical journals [21] not indexed in PubMed [18] or Embase [19]. These unindexed journals are published online in Mongolian or English by the Mongolian Association of Medicine [21]. We will also manually search the archives of the Mongolian National Conference on “Current Topics of Virology” [18]. Lastly, the literature search will be supplemented by checking references of the included reports and identified reviews. We will use broad search criteria with no language nor time restrictions.

### Study selection and data extraction

The titles and abstracts, and consequently the full-texts of identified unique reports from PubMed [18] and Embase [19], will be independently screened for relevance by two reviewers. The titles and abstracts, and consequently the full-texts of identified unique reports, in Mongolian will be screened for relevance by two Mongolian native-speaker reviewers. Discrepancies will be discussed and resolved by the study team. Non-eligible reports will be excluded, and the reasons for their exclusion will be recorded.

Extraction of relevant data will be conducted using a standardized piloted form. Extraction of relevant data from reports identified through PubMed [18] and Embase [19] will be conducted by one reviewer and 25% of the measures will be checked for correctness by another reviewer. Extraction of relevant data from reports in Mongolian will be extracted by two Mongolian native-speaker reviewers. We will extract data on study and report characteristics, participant characteristics, and primary outcomes of interest. When available, secondary outcomes of interest will be also extracted. When reported, we will extract outcome measures estimated from separate subsets of stratified data.

### Assessment of risk of bias in individual studies

The quality of individual studies will be examined at the study and outcome levels.

Regarding the risk of bias (ROB) assessment at study-level, studies will be classified based on an adaptation of the Cochrane principles [22]. This adaptation of the ROB assessment is also based on the methodology of other systematic reviews of HCV prevalence [11–16]. We will use three quality domains, namely, sampling methodology, HCV infection ascertainment, and response rate, as having a low, high, or unclear ROB. A ROB is considered low if (1) sampling is probability-based, (2) HCV infection is ascertained by biological assays, or (3) response rate is ≥80%. Studies with missing information for a specific domain will be classified as having unclear ROB for that specific domain.

Regarding the ROB assessment at outcome-level [23], an HCV-Ab prevalence measure will be considered as having good precision if the number of HCV tested individuals is greater than a calculated minimum sample size. This calculated sample size will be derived from the binomial (Clopper-Pearson) confidence interval formula [24].

### Qualitative data synthesis

Extracted outcome measures (HCV-Ab incidence, HCV-Ab prevalence, HCV RNA prevalence, HCV genotypes/subtypes, and risk factors) will be reported according to the study population’s risk of acquiring HCV infection (likelihood of being exposed to HCV: (1) populations at low-risk: these include populations such as the general population, blood donors, pregnant women, and healthy adults and children. (2) Populations at intermediate-risk: these include populations such as healthcare workers, medical students, patients with sexually transmitted diseases, HIV infected persons where the main suspected mode of transmission is sexual, female sex workers, men who have sex with men, homeless people. In addition, as mobile men in Mongolia are considered to be at a greater risk for HIV and STIs due to a greater probability of having casual sex partners and/or having sex with female and/or male sex workers [25], mobile men and traveling traders will be also included in the category of populations at intermediate-risk. (3) Populations at high-risk: these include populations such as people who inject drugs and patients with hemophilia, hemodialysis, thalassemia, and/or multiple transfusions, and HIV infected persons where the main suspected mode of transmission is parenteral. (4) Special clinical populations: these include patients with specific medical conditions that could be related to HCV infection such as chronic liver disease, acute viral hepatitis, liver cirrhosis, hepatocellular carcinoma, and glomerulonephritis. Special clinical populations also include patients referred for HCV testing. Additionally, special clinical populations include other clinical populations who could have been exposed to HCV in clinical settings due to the specific nature of HCV epidemiology in Mongolia; but at uncertain level of exposure making it difficult to classify them under the former three at-risk population categories. This category will include also the immune compromised populations other than HIV/AIDS patients. 5) Mixed populations: these include a mix of at least two of the at-risk population categories indicated above.

### Quantitative data synthesis

#### Meta-analysis

Regarding the quantitative synthesis, we will pool primary measures including HCV Ab-incidence, HCV-Ab prevalence, and HCV genotypes/subtypes among different at-risk populations, and secondary outcomes as relevant such as RNA prevalence. Outcome measures with sample sizes of at least 25 participants will be eligible to be included in the meta-analyses. Outcome measures estimated from stratifications by subpopulation, and/or sex, and/or study period, and/or region, and/or age, will be prioritized for inclusion from each given included report. As such, if a report provides regional and national outcome measures for the same population, we will include the regional measures. Binomial method [24] will be used to compute the 95% confidence interval (95% CI) for each outcome measure. We will pool HCV-Ab prevalence by at-risk population category (low-risk, intermediate-risk, high-risk, and special clinical populations). We will also pool HCV RNA prevalence in HCV-Ab positive individuals from the general population that is the prevalence of chronic HCV infection among the HCV-Ab positive general population. Meta-analyses will be conducted for each specific category if at least three outcome measures meet the eligibility criteria for the quantitative synthesis.

All meta-analyses will be conducted using random-effects models, to account for expected heterogeneity in outcome measures across studies. Outcome measures will be weighed by the inverse-variance of the double-arcsine transformed proportions [26], according to the method described by DerSimonian and Laird [27].

#### Assessment of the heterogeneity across studies

In order to assess heterogeneity in outcome measures across studies, forest plots will be inspected visually and Cochran’s *Q* test will be conducted [28]. A two-sided Cochran’s *Q* test *p* value of <0.10 will be considered as significant. *I* ^2^ heterogeneity measure and its 95% CI will be calculated to assess the magnitude of between-study variation that is due to heterogeneity across studies rather than chance [29]. The 95% prediction interval will be also estimated to describe the distribution of true effect size (HCV prevalence) around the pooled mean estimate [29, 30].

In order to explore the variation in pooled HCV-Ab prevalence and heterogeneity in risk of exposure between subgroups, we will conduct subgroup meta-analyses and meta-regressions as warranted by the extracted data. We will pool HCV-Ab prevalence by at-risk subpopulation (such as blood donors, healthcare workers, individuals engaging in risky sexual behaviors, special clinical patients without liver disease, patients with acute hepatitis, patients with chronic liver disease, patients with liver cirrhosis, and patients with hepatocellular carcinoma), time period, study location or region, sex, and age group. Comparisons between subgroups will be conducted using the *Q* test for subgroup differences [30]. *Q*_between subgroups_ test *p* value of <0.05 will be considered as significant.

We will investigate sources of heterogeneity between studies using meta-regression [22]. Potential effect modifiers will be: subpopulations (cited above), sources of reports (journals indexed in PubMed [18] and Embase [19], conferences indexed in Embase [19], and journals and conferences unindexed in PubMed [18] and Embase [19]), study sites (such as community, blood bank, clinical setting, school, and mixed sites), and biological assays (such as enzyme immune assay, enzyme-linked immunosorbent assay, hemagglutination assay, chemiluminescent enzyme immunoassay, and multiplex HCV serology assay). These factors will be entered into univariable models and included in the final multivariable model if the *t* test *p* value is <0.10.

All data analyses will be conducted in R v.3.1.1. (Vienna, Austria) [31] using the meta [32] and metafor [33] packages.

#### Age-specific and age-adjusted HCV-Ab prevalence and age-adjusted chronic HCV infection prevalence

Pooled age-specific HCV-Ab prevalence trend among the general population will be assessed using a polynomial regression model. Multiple R-square will be estimated to evaluate how well the model fits the pooled age-specific HCV-Ab prevalence trend. Analysis of variance will be conducted to identify which order of the polynomial regression model should be selected. The significance of the observed trend will be assessed by the *F* test with a significance threshold at 0.01.

Using the de facto population distribution by age group provided by the Census 2010 as extracted from the database of the Population Division of the United Nations Department of Economic and Social Affairs [34] and using our estimates of pooled age-specific HCV-Ab prevalence among the general population, we will calculate the total number of individuals exposed to HCV infection in Mongolia and the age-adjusted HCV-Ab prevalence among the population of Mongolia. By assuming pooled mean HCV RNA prevalence among HCV-Ab positive individuals from the general population is the same in all age groups, we will also estimate the number of chronically infected individuals by age group and the age-adjusted prevalence of chronic HCV infection among the population of Mongolia.

#### Publication bias

Regarding publication bias assessment, we will use funnel plots to explore small-study effect on the pooled estimates. A minimum of ten studies will be required for this assessment. We will produce funnel plots of log (odds proportion) against study size [35], with *odds proportion* defined as *prevalence/(1-prevalence)*. In order to test the asymmetry in the funnel plots, we will conduct Peter’s tests [35].

#### Confidence in cumulative evidence

As in an earlier systematic review [23], we will use a narrative justification for the quality of evidence based on the Cochrane handbook [22]. Our confidence in evidence will be based on the number of high quality studies conducted at the national level. High quality will be determined by having low ROB in the three domains. Mongolia will be classified in one of the following categories according to the strength of evidence: (1) no evidence: no data identified; (2) poor evidence: poor quality of outcome measures; (3) limited evidence: the number of outcome measures is small but of reasonable quality; (4) good evidence: the number of outcome measures is small but of good quality; or (5) conclusive evidence: sufficient outcome measures of good quality.

## Discussion

This systematic review and meta-analysis will determine HCV-Ab prevalence and its distribution, RNA prevalence, HCV-Ab incidence and HCV genotypes/subtypes in Mongolia among different at-risk populations. The methodology to be used in the proposed study will ensure a robust synthesis of available data. The ultimate aim of our work is to provide empirical evidence necessary for researchers, policy-makers, and public health stakeholders to set research, policy, and programming priorities for HCV prevention, control, and eventually elimination in Mongolia.

## Additional file

Additional file 1: PRISMA-P 2015 Checklist. (DOCX 30 kb)

## Abbreviations

95%CI: 95%confidence interval
Ab: Antibody
HCV: Hepatitis C virus
PRISMA2009: Preferred Reporting Items for Systematic Reviews and Meta-Analyses
PRISMA-P2015: PRISMA for systematic review protocols
*p* value: Probability value
RNA: Ribonucleic acid
